# Bibliographical

**Published:** 1886-01

**Authors:** 


### BIBLIOGRAPHICAL.

DENTAL BIBLIOGRAPHY. *A standard reference list of Books of Dentistry, published throughout the world, from* 1536 *to* 1885; *arranged Chronologically, and supplemented with a complete cross-reference to authors.* By C. GEO. CROWLEY. Philadelphia: The S. S. White Dental Manufacturing Company. 1885.

Many dentists have bewailed the paucity of dental literature. How many books does the reader imagine are catalogued in this volume? No less than *two thousand and forty-seven* separate works and editions. Nor does this include the innumerable pamphlets and monologues that have been issued all over the world. Surely, we make a better showing than most of us had thought possible.

The book shows the most intimate acquaintance, on the part of the compiler, with our literature. It also indicates remarkable bibliographical intelligence, and great industry. We have known Mr. Crowley as a bibliophile, and have often had occasion to consult him concerning books of which no one else seemed to know anything, and have never found his knowledge at fault. Had we been called upon to select the man best qualified for this bibliographical task, we should unhesitatingly have pointed out Mr. Crowley, and his work would have justified the choice.

The work is divided into five departments or sections. Section I contains books published in Germany, Austria, Holland, Norway, Sweden, Denmark, and Switzerland (German); Section II books published in France, Belgium, and Switzerland (French); Section

III books published in Spain and Italy; Section IV books published in Great Britain and Ireland; Section V books published in America.

The reader may be curious to know which language has proved most prolific of dental books. Section I, (German) has furnished 752 works; Section II, (French) 710; Section III, (Spanish and Italian) 46; Section IV, (Great Britain) 230; Section V, (America) 309. It will thus be seen that Germany leads in our literature, but is closely followed by France, and either has given us more books than all the English speaking nations, while the Spaniards and Italians are far in the rear.

Samuel S. Fitch had, to his "System of Dental Surgery," appended a bibliography, and names about 350 different works. (Our edition is the second, 1835.) Maury (Treatise on the Dental Art, American edition, 1843), names about 250 different authors, and the translator adds about 40 more. Paul B. Goddard (Anatomy, Physiology and Pathology of the Human Teeth, 1844), enumerates 287 different authors. J. E. Dexter (History of Dental and Oral Science in America, 1876), enumerates 70 different American works. It will be seen that neither of these authors has attempted to make a complete bibliography, nor will their lists at all compare with that under notice. The publishing of a complete and correct list of its books to date marks an important era in the history of any profession, for it is only by its aid that an entire knowledge of the advancement as a profession can be determined. Such a work grows more valuable with time, and any dentist who makes pretensions to a knowledge of his professional literature will have a copy. If he does not possess the list of books belonging to his calling, when it is so easily secured, he can have no claims to literary knowledge. This book is admirable in every way, and in every department the work seems to be thoroughly done.

The profession owes a debt of gratitude to Mr. Crowley for what he has accomplished. It will hardly be expected that the sales will sufficiently remunerate him for the great expenditure of time that the necessary researches have demanded. The First District Dental Society has already conferred upon him an honorary membership. We hope that some one of our institutions of learning will recognize his bibliographical attainments and the devotion which he has shown to the profession, by some fitting honorary distinction that will show that we are not ungrateful.

DENTAL MEDICINE. *A Manual of Dental Materia Medica and Therapeutics, for Practitioners and Students.* By FERDINAND J. S. GORGAS, A. M., M. D., D. D. S. Second edition. Revised and enlarged. Philadelphia: P. Blakiston, Son & Co. 1885.

In the last volume of this journal we reviewed the first edition of this work, and now we are called upon to notice the second, It is a very unusual event in the history of any medical book, when the second edition is so soon called for. When the first appeared we predicted that it would meet with favor, and the unusual sales have verified the not very hazardous forecast. The second edition is a considerable improvement upon the first. There are always minor particulars in which a book may be improved, and these cannot usually be detected until the work has been for a time in the hands of the public. Sixty-eight pages have now been added, and the whole text revised. Short as has been the time since the first edition, there were already a number of new remedies offered to the profession, all of which, we believe, have received due notice. Cocaine, Peroxide of Hydrogen, Papain, Resorcin, Eugenol, Naphthalin, Chinoline, Ol. Sanitas, with a number of other fresh aspirants to favor are considered. There is also a new chapter on Inflammation of Mucus Surfaces, while that upon Diagnosis has been rewritten and considerable valuable matter added to it.

Gorgas' Dental Medicine has already taken its place near the head of our professional literature. It has, we believe, no equal in the English language, if in any other, as a text-book on dental medicine, and we shall look to see it adopted in all countries, where there is dental teaching, as the standard work on dental Materia Medica and Therapeutics.

DISEASES OF THE TONGUE. BY HENRY T. BUTLIN, F. R. C. S., *Assistant Surgeon and Demonstrator of practical Surgery and Diseases of the Larynx, St. Bartholomew's Hospital; lately Erasmus Wilson Professor of Pathology at the Royal College of Surgeons.* Illustrated with chromo-lithographs and engravings. Philadelphia: Lea Brothers & Co. 1885.

At first thought it might be believed that the devotion of a whole book to the consideration of so small an organ as the tongue would make but unprofitable reading. Yet here is a work of four hundred and fifty closely printed pages, not one of which could well have been omitted. When we reflect that the tongue presents an index of the condition of the whole digestive tract, and that upon its surface may be traced some indication of almost every disease that attacks man, and that its deeper tissues are subject to many pathological changes, the significance of such a work may be partially understood. The one under notice has twenty-five chapters, and some of the subjects of which it treats are Accidents, Congenital Defects, Discolorations, Inflammations, Eruptions, Indentations, Excoriations, Furrows, Fissures, Ulcers, Patches and Plaques, Nodes and Nodules, Smooth Patches, Atrophy, Hypertrophy, Cysts, Salivary Calculus, Tumors, Cancers, Operations, Parasitic Affections, and Nervous Affections of the Tongue. It has also a list of works and papers referring to diseases of the tongue, and a copious index.

Every oral surgeon should be thoroughly acquainted with this organ, and dentists especially, who are constantly engaged in the treatment of oral diseases, cannot afford to remain in ignorance of the subjects treated of in this book. It is a duty which all such owe to their patients to become thoroughly familiar with the tissues of the mouth, of which the tongue is the most important. We could not refer them to a work that would prove of greater interest to them, and we unhesitatingly recommend it to every progressive dentist. A thorough study of its contents by members of our societies would enlarge the field of discussion, and add greatly to the interest of the meetings.

ESSENTIALS OF VACCINATION. *A compilation of facts relating to vaccine inoculation and its influence in the prevention of Small-Pox.* By W. A. HARDAWAY, M. D., Professor of Diseases of the Skin in the Post Graduate Faculty of the Missouri Medical College, St. Louis. St. Louis: J. H. Chambers & Co. 1886.

This book is timely. The intense interest that has been developed, concerning vaccination, by the epidemic of small-pox in Canada, and the occurrence of the vaccination riots in Montreal, has directed the attention of the whole world to the only real prophylactic with which we are acquainted. There has always been in existence a class of men who, upon principle, disputed what all the rest of the world believed. There has not lived a great leader of the people, from Moses to Washington, whose very existence they have not denied. So it is not astonishing that the virtues of vaccination have been disputed. Upon the truth of the general theory of inoculation rest the hopes of the world for the prevention of many infectious disorders. If vaccination be not preventive of small-pox, Pasteur's experiments are vain.

Every one will, therefore, desire to know the history of, and the facts relating to, vaccination, and that is what this book professes to give. The nature of the virus and the changes wrought by it are all intelligently considered. The work will prove of great interest to all intelligent men.

A SERIES OF QUESTIONS PERTAINING TO THE CURRICULUM OF THE DENTAL STUDENT. BY FERDINAND J. S. GORGAS, M. D., D. D. S., of the University of Maryland. Baltimore: Wm. K. Boyle & Son. 1885.

For the dental student such a work is invaluable. It not only gives proper direction to his studies, but it teaches him conciseness and preciseness. In preparing for examination it becomes an essential. Nor is it alone the student who will find it of value. The practitioner, who would retain in his memory the teachings of his college days, and who would keep abreast the march of progress, would do well to take up a chapter occasionally, and ask of himself the questions which it contains. If he be not at times astonished that he should have forgotten so much, shocked at his own ignorance, and thereby spurred on to a renewed application to his text-books, his experience will be different from that of most men.

The many years which Prof. Gorgas has spent as a teacher in our dental colleges especially qualifies him for the preparation of such a work as this. Nor is it the training of experience alone that he brings to the task. He has the broad views, the general culture, and the discriminating mind that are so necessary to the successful teacher, and all these qualities are amply exhibited in this excellent series of questions, which covers almost the whole field of dental research.

THE CINCINNATI MEDICAL AND DENTAL JOURNAL. Monthly. M. A. Spencer & Co., Publishers, Cincinnati, Ohio. $1.00 per Annum.

This is a new aspirant for dental favor. It starts out, as did the INDEPENDENT PRACTITIONER originally, with a Medical and Dental department. The former is under the charge of A. B. Thrasher, A. M., M. D., and the latter, of Frank W. Sage, D. D. S. Both the editors are accomplished writers, and together they present a journal of interest. But our experience, as one of the editors of a journal with both a medical and dental department, has taught us that each suffers at the hands of the other. Thirty-two pages are not too much for the presentation of a fair resumé of either. A journal should have a definite aim, and we do not think it can successfully cover the ground of both general and special practice. But we hope that the *Medical and Dental Journal* will disprove these impressions, and we wish it the most abundant success. The experiment is worth another and more perfect trial.

TRANSACTIONS OF THE AMERICAN DENTAL ASSOCIATION. *Twenty-fifth Annual Session, held at Minneapolis, Minn., August,* 1885. Philadelphia: The S. S. White Dental Mfg. Co.

The annual volume comes to us much improved in appearance this year, as it is substantially bound. The papers are all given in full, and some of them are of great value, but the discussions, it seems to us, are not as complete as in some former issues. Perhaps this is an improvement also, for there is very much said at the meetings which is not only of no moment, but the publication of which would be actually detrimental to the volume. Its typographical appearance, like that of all the books published by this house, is very neat indeed. It presents indications of great care in the proof-reading and press-work.

TRANSACTIONS OF THE TEXAS STATE MEDICAL ASSOCIATION. *Seventeenth Annual Session, held at Houston, Texas, April,* 1885.

We are under obligations to Dr. F. E. Daniels, chairman of the Committee on Publication, and editor of that sprightly monthly, *Daniels' Medical Journal,* for a copy of this book. It forms a volume of 430 pages, and is beautifully bound and executed. That much is gained by having a live journalist at the head of the Publication Committee.

DENTOLOGIA. *A Poem on the Diseases of the Teeth, and their Proper Remedies.* By SOLYMAN BROWN, A. M.

We are indebted to the son of the author, Dr. E. Parmly Brown, for a copy of this beautiful poem. It is contained in the *Semi-Annual Dental Expositor* for May, 1852, copies of which are now exceedingly rare, and hard to be obtained. If we had but the space to spare we would gladly quote passages from it, to show the easy flowing rhythm, and the luxuriant imagery of this, the first of dental poems.

PHYSICIANS' VISITING LIST FOR 1886. Philadelphia: P. Blakiston, Son & Co.

This is the thirty-fifth year of the publication of this work. Little more need be said in its favor. It contains calendar, list of poisons and antidotes, dose tables, Marshall Hall's and Sylvester's methods of producing artificial respiration, with illustrations, diagram for diagnosing diseases of the heart, lungs, etc., and much other valuable information.

THE DENTAL ECLECTIC.

This is a new bi monthly journal, devoted to dentistry, published by H. L. Willard, of the Knoxville Dental Depot, and edited by S. S. Willard, D. D. S. The first numbers present a good appearance, and appear to be well edited. Fifty cents per year.

*On the Measurement of the Degree of Anæsthesia Produced by Cocaine.* By Lucien Howe, M. R. C. S., Buffalo, N. Y. Reprinted from the *Archives of Ophthalmology.*

*Effect of Cocaine upon the Healing of Wounds.* By Lucien Howe, M. D., Buffalo, N. Y. Reprinted from the Transactions of the Medical Society of the State of New York.

These two pamphlets give the records of exhaustive experiments upon animals, and are conclusive in their results. Dr. Howe is an indefatigable student. All his experiments are conducted in a strictly scientific manner, and the conclusions carefully tabulated.

*Pure Cultures for Bacteria.* By Albert Haupt, M. D., Chenmitz, Saxony. A paper read before the American Institute of Homœopathy, at St. Louis, June, 1885.

*Iritis.* Its relation to the Rheumatic Diathesis, and its treatment. By Charles J. Landy, A. M., M. D. Reprinted from *The Physician and Surgeon.*

*The Surgical Treatment of Cysts of the Pancreas.* By N. Senn, M. D. Reprinted from the *Journal of the American Medical Association.*

*Observations upon the Mutual Relations of the Medical Profession and the State.* President's Address, delivered before the Michigan State Medical Society. By Donald Maclean, M. D.

*Avena Sativa in the Treatment of Opium Addiction.* A therapeutical fraud, a delusion and a snare. By J. B. Mattison, M. D., Brooklyn, N. Y. Reprinted from the *Medical Bulletin.*

